# Biochar-Based Single-Atom Cobalt Catalyst for Efficient Thermal Decomposition of Ammonium Perchlorate: Preparation, Performance and Mechanism

**DOI:** 10.3390/ijms27135964

**Published:** 2026-07-02

**Authors:** Yixin Liu, Xiaolin Tang, Bin Zhang, Yuming Zhou, Junyu Li, Zeyu Zheng, Yifu Zhang, Yanfen Huang, Chi Huang

**Affiliations:** 1 College of Chemistry and Molecular Sciences, Wuhan University, Wuhan 430072, China; 2024282030194@whu.edu.cn (Y.L.); winters@whu.edu.cn (X.T.); yumingzhou@whu.edu.cn (Y.Z.); junyuli@whu.edu.cn (J.L.); zhengzeyu@whu.edu.cn (Z.Z.); 2 System Design Institute of Hubei Aerospace Technology Academy, Wuhan 430040, China; zhangbin8110@163.com; 3 Hubei Key Laboratory of Radiation Chemistry and Functional Materials, School of Nuclear Technology and Chemistry & Biology, Hubei University of Science and Technology, Xianning 437100, China; 4 School of Chemistry and Chemical Engineering, Wuhan University of Science and Technology, Wuhan 430081, China; huangyanfen@wust.edu.cn

**Keywords:** single-atom catalyst, biomass carbon, cobalt-based catalyst, thermal decomposition, ammonium perchlorate

## Abstract

The thermal decomposition performance of ammonium perchlorate (AP) is a key factor in regulating the combustion of composite solid propellants, and its catalytic decomposition process is considered a typical multiphase catalytic process. The exposure of catalytic centers during multiphase catalysis is a key factor affecting catalytic performance. In response to the problem of low atomic utilization efficiency of traditional metal oxide catalysts, this study successfully prepared nitrogen-doped carbon-supported single-atom cobalt catalyst (SACo-PC-X) using the “zinc volatilization pore formation and nitrogen anchoring” method with inexpensive biomass as the precursor. Aberration-corrected transmission electron microscopy, together with XPS and XRD analyses, suggests that Co species are predominantly stabilized in an atomically dispersed Co-N_x_ configuration. This catalyst exhibits excellent catalytic performance for the thermal decomposition of AP, significantly reducing its high-temperature decomposition temperature from 433.5 °C to 322.7 °C (The cobalt content in the system is less than 0.2%). Gas studies have shown that Co-N_x_ sites efficiently accelerate the oxidation process of NH_3_ by promoting electron transfer, resulting in a significant increase in the proportion of N_2_O gas. This work not only provides an efficient and stable new catalyst for AP decomposition, but also offers new ideas for designing energetic material decomposition catalysts at the atomic level.

## 1. Introduction

Solid propellants function as the cornerstone of propulsion systems for missiles, rockets, and spacecraft. Their combustion performance is intrinsically governed by the thermal decomposition behavior of the oxidizer. Ammonium perchlorate (AP) remains one of the most widely used oxidizers in composite solid propellants for these applications because of its high oxygen content and low cost [1,2]. The thermal decomposition of AP is a complex, multiphase process involving solid and gaseous intermediates, typically proceeding through two distinct stages: low-temperature decomposition (LTD, ~300 °C) and high-temperature decomposition (HTD, ~400 °C) [3,4]. However, the utility of pure AP is hindered by its elevated decomposition temperature and sluggish reaction kinetics. These limitations significantly constrain enhancements in the burning rate of the propellant. Consequently, the development of high-efficiency catalysts capable of lowering the thermal decomposition temperature and accelerating energy release has emerged as a focal point of enduring research within the field of energetic materials [5,6,7].

Previous studies have shown that AP thermal decomposition can be promoted by several types of catalyst systems, including transition-metal oxides, carbon-supported composites, and hybrid nanostructures [8]. Among transition-metal oxide catalysts, Co_3_O_4_ nanowire arrays, porous Co_3_O_4_/C composites, and Co_3_O_4_@ZnO heterostructures have been reported to enhance AP decomposition through morphology regulation, porous-structure construction, and interfacial electron-transfer promotion [9,10,11]. In addition, MOF-derived catalysts provide an effective route for constructing highly dispersed metal/carbon composite structures; for example, a ZIF-67-derived Co@NC catalyst reduced the HTD peak of AP by 100.5 °C, decreased the apparent activation energy by 82.0 kJ·mol^−1^, and increased the heat release to 2.9 times that of pure AP [12]. Carbon-supported composite catalysts have also been developed to combine the advantages of porous carbon frameworks and active metal species, as demonstrated by 3D hierarchically ordered porous carbon-confined Fe_2_O_3_ nanoparticles and nitrated graphene oxide systems [13,14]. Moreover, 3 wt% Co_3_O_4_/graphene composite decreased the decomposition temperature of AP by 95.2 °C and eliminated the low-temperature decomposition peak [15]. Graphitic carbon nitride was also reported as a metal-free catalyst, decreasing the AP decomposition temperature by 70 °C, reducing the activation energy from 216.0 to 119.8 kJ·mol^−1^, and increasing the apparent heat release from 574.2 to 1362.6 J·g^−1^ [16]. Further improvement was achieved by directly growing CuO nanorods on g-C_3_N_4_, which reduced the AP decomposition temperature by up to 105.5 °C and transformed the two-step decomposition process into a single main exothermic process [17]. These representative studies indicate that increasing the exposure of active sites and improving interfacial electron/mass transfer are key strategies for enhancing AP thermal decomposition. Meanwhile, AP decomposition itself is generally considered to involve distinct low-temperature and high-temperature stages, together with coupled condensed-phase and gas-phase reactions [18]. Therefore, both the current catalyst state of the art and the general AP decomposition process should be considered when evaluating newly developed AP decomposition catalysts.

Although traditional catalysts, such as transition metal oxides (TMOs; e.g., Fe_2_O_3_, Co_3_O_4_, CuO) and their derivatives, are effective in lowering the thermal decomposition temperature of AP, their application is restricted by intrinsic limitations [19]. It has been demonstrated that TMO catalysts are prone to agglomeration during use, which limits their efficacy. Furthermore, the size of the oxidant itself also limits the exposure of its internal metal active sites [20]. Moreover, metal atoms confined within the bulk lattice are inaccessible for surface reactions, resulting in low atomic utilization efficiency. Furthermore, the poor interfacial compatibility between inorganic oxides and the AP/binder matrix poses a risk of compromising the mechanical integrity of the composite propellant [21]. 

Recently, single-atom catalysts (SACs) have emerged as a frontier in electrocatalysis and photocatalysis, owing to their near-unity atom utilization, structurally uniform active sites, and unique electronic properties [22,23,24]. These catalysts feature isolated metal atoms anchored onto specific supports. Notably, metal-nitrogen (M-N_x_) moieties embedded within nitrogen-doped carbon matrices are widely recognized as highly efficient catalytic centers in heterogeneous catalysis [25,26]. More importantly, atomically dispersed catalysts have recently begun to be introduced into AP thermal decomposition catalysis. Zhang et al. [27] reported single Cu atoms anchored energetic COFs as combustion catalytic promoters, and the highly accessible Cu atomic sites promoted the rapid and concentrated thermal decomposition of AP. Zhu et al. [28] developed copper-enriched polydopamine single-atom catalysts for the synergistic catalysis of AP and Ammonium Dinitramide (ADN) decomposition, showing that atomically dispersed Cu sites exhibited advantages over conventional CuO nanoparticles. These studies demonstrate that isolated metal centers can regulate AP decomposition at the atomic level. Nevertheless, the reported single-atom catalytic systems for AP decomposition still mainly rely on deliberately designed energetic frameworks or polydopamine-confined structures, whereas the construction of efficient AP decomposition catalysts from low-cost biomass waste remains much less explored. Carbonaceous supports not only ensure the stable dispersion of metal atoms but also exhibit superior thermal and electrical conductivity—properties that are vital for managing the rapid heat release associated with the vigorous exothermic decomposition of AP. Furthermore, synthesizing SACs from biomass precursors offers a sustainable and cost-effective strategy; the inherent abundance of heteroatoms (e.g., N and O) in biomass facilitates the anchoring and stabilization of metal centers, while the direct utilization of rice waste further contributes to waste valorization and circular-economy-oriented catalyst design [29,30,31].

Against this background, the present study focuses on low-cost biowaste recycling and the direct utilization of discarded rice waste as a carbon/nitrogen precursor for constructing Co-N_x_ single-atom sites for AP decomposition. Unlike our previously reported MOF-derived Co single-atom catalyst based on a ZnCo-ZIF precursor [32], this work uses discarded rice waste as the starting carbon/nitrogen source, which has the advantages of low cost, easy availability of raw materials, tendency towards pore formation and Co loading, and controllable morphology. Then, hydrothermal carbonization, ZnCl_2_ activation, and thermal capture of Co species are carried out to construct atomically dispersed Co-N_x_ sites on biomass-derived porous carbon. Although the AP decomposition peak temperature achieved in this work is slightly higher than that of the previously reported MOF-derived catalyst, the present strategy demonstrates a sustainable waste-to-catalyst route for preparing efficient AP decomposition materials from low-cost biowaste. Therefore, the main significance of this work lies not in pursuing the lowest decomposition temperature, but in broadening the precursor scope of Co single-atom catalysts and highlighting the value of waste-biomass valorization in energetic-material catalysis.

## 2. Results and Discussion

### 2.1. Synthesis and Characterization of SACo-PC

**Figure 1:**
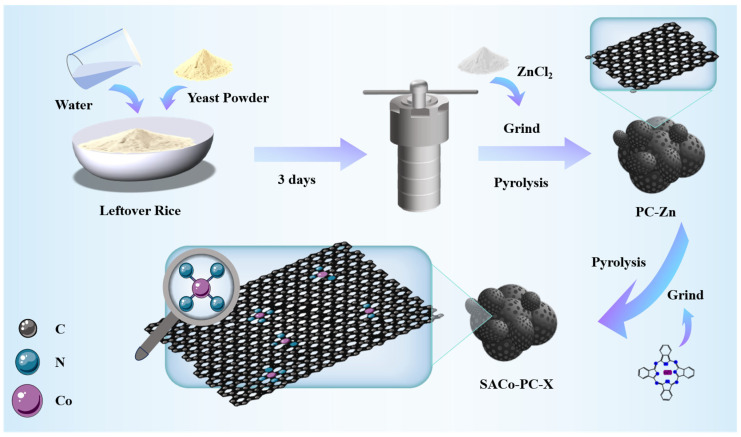
Schematic diagram of the formation procedure of SACo-PC-X.

In this work, we used leftover cooked rice collected from a school cafeteria as the raw material and carried out a water-thermal reaction and high-temperature calcination to generate the precursor SACo-PC-X for catalyzing AP (Figure 1). The hydrothermal treatment itself was not intended as a completely new synthetic protocol, but was adapted from the general hydrothermal carbonization strategy widely used for converting wet biomass into hydrochar precursors. First, the fermentation of waste rice triggered the hydrolytic degradation of starch and proteins into glucose and amino acid intermediates. Under hydrothermal conditions 180 °C, these monomers underwent complex dehydration and polymerization-aromatization reactions, yielding a nitrogen-rich hydrochar framework where nitrogen species from yeast were intrinsically incorporated into the carbonaceous matrix.

Subsequently, ZnCl_2_ was introduced during the activation step to assist the formation of porous carbon. Previous studies have shown that ZnCl_2_ can act as a chemical activating agent for biomass-derived carbon materials, promoting dehydration, aromatization, and pore development during high-temperature carbonization [33,34]. In this work, the ZnCl_2_-assisted activation process, together with the volatilization of Zn-containing species during pyrolysis, is expected to contribute to the formation of the rough and porous carbon framework observed in the SACo-PC-X samples. Such a porous structure can increase the accessibility of the carbon surface and provide defect-/nitrogen-containing anchoring environments that are favorable for the subsequent stabilization of Co species.

Finally, the SACo-PC-X was finalized via a “thermal-trapping” mechanism during the 800 °C secondary pyrolysis. Additional SEM images of PC-Zn and SACo-PC-X are provided in Figure S1 (Supporting Information). Cobalt phthalocyanine (CoPc) served as the metal precursor, which underwent structural decomposition to release mobile cobalt species. The resulting Co species interacted with the pre-existing nitrogen-containing sites and defects in the PC-Zn matrix. Such strong metal–support interactions are expected to stabilize highly dispersed Co species and suppress their sintering into larger particles [35]. 

**Figure 2:**
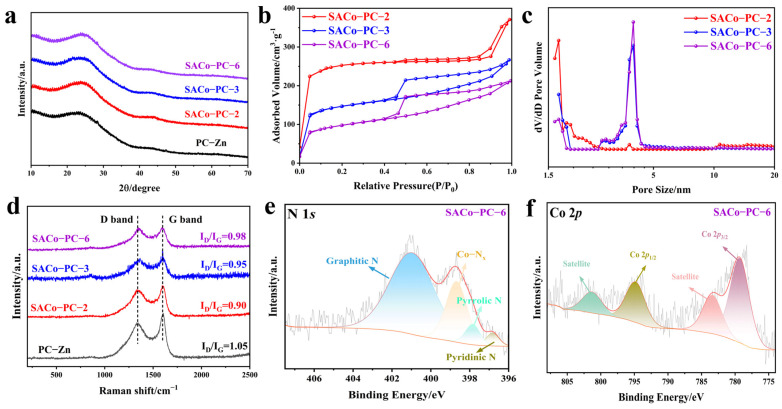
Structural characterization of PC-Zn and SACo-PC-X: (**a**) XRD graph of PC-Zn and SACo-PC-X; (**b**) N_2_ sorption isotherms of SACo-PC-X at 77 K; (**c**) Apparent pore-size distribution of SACo-PC-X derived from N_2_ sorption data; (**d**) Raman graph of PC-Zn and SACo-PC-X; (**e**) XPS N 1*s* graph of SACo-PC-6; (**f**) XPS Co 2*p* graph of SACo-PC-6.

To investigate the crystalline phases of the cobalt species within the carbon matrix, XRD measurements were performed on the synthesized samples. As illustrated in Figure 2a, two broadened diffraction peaks centered at 2θ ≈ 24° and 43° are observed for both the PC-Zn precursor and the SACo-PC-X series with varying cobalt loadings. These reflections can be assigned to the (002) and (100) planes of disordered carbon and are close to the characteristic positions of graphitic carbon (PDF No. 41-1487), consistent with previous reports on biomass-derived porous carbon materials [36]. Their broadened features indicate a low degree of graphitization and a turbostratic stacking structure.

Crucially, even for the SACo-PC-6 specimen featuring the highest nominal cobalt loading, no characteristic diffraction reflections ascribable to metallic Co or cobalt oxides/hydroxides were discernible in the XRD patterns. Typically, crystalline domains of metal nanoparticles (larger than 3–5 nm) would manifest as sharp diffraction peaks. Consequently, this “crystallographically silent” behavior provides preliminary evidence that the Co species are highly dispersed across the carbon matrix, with particle dimensions likely falling below the resolution limit of XRD, implying the predominance of sub-nanometric clusters or isolated single atoms. Complementary ICP-OES analysis further showed that the actual Co loadings of SACo-PC-2, SACo-PC-3, and SACo-PC-6 were 1.46 wt%, 2.48 wt%, and 3.89 wt%, respectively. The ability to maintain such an atomic-level dispersion without aggregation at this high loading level is ascribed to the robust anchoring effect exerted by the abundant nitrogen heteroatoms derived from the biomass precursor, which effectively immobilize the metal atoms.

Pore structure analysis of the SACo-PC-X series was performed using N_2_ adsorption–desorption measurements (Figure 2b,c), and the detailed porous properties of the PC-Zn precursor are also provided in Figure S2 [37] (Supporting Information). The N_2_ adsorption–desorption isotherms of the SACo-PC-X series can be classified as type IV(a) isotherms with H3 hysteresis loops according to the IUPAC technical report [38], indicating the presence of mesoporosity together with slit-like or interparticle pore features. The pore-size distribution shown in Figure 2c was derived from the N_2_ sorption data using the NLDFT equilibrium model. Considering that cavitation during N_2_ desorption at 77 K may introduce artefactual features in the narrow mesopore range, the apparent pore-size concentration near 3–5 nm should be interpreted with caution rather than taken as definitive evidence of a uniquely narrow pore-size distribution. Therefore, the N_2_ sorption results mainly support the mesoporous nature of the SACo-PC-X materials and the existence of pore channels favorable for mass transfer during AP decomposition. Such accessible porous structures are beneficial for catalysts applied in AP thermal decomposition. Specifically, this architecture can facilitate the exposure of accessible active sites and provide diffusion pathways for gaseous reactants and intermediates, such as NH_3_ and HClO_4_. Consequently, the improved mass transport is expected to alleviate the local accumulation of gaseous intermediates during the vigorous decomposition reaction.

Raman spectroscopy in Figure 2d was employed to further probe the degree of graphitization and structural disorder within the carbonaceous framework. All synthesized samples exhibit two distinct characteristic peaks located at approximately 1350 cm^−1^ and 1590 cm^−1^, corresponding to the defect-induced D band and the graphitic G band, respectively. The intensity ratio of the D band to the G band (I_D_/I_G_) varies slightly from 1.05 for the PC-Zn precursor to 0.98 for the SACo-PC-6 catalyst. The slight decrease in the I_D_/I_G_ ratio suggests that Co incorporation does not introduce a large number of additional structural defects. Combined with the XPS evidence for Co-N_x_ species, this result is more reasonably interpreted as being consistent with the utilization of pre-existing defect-/N-containing anchoring sites in the carbon framework.

Deconvolution of the high-resolution N 1*s* spectrum (Figure 2e) reveals four nitrogen species, including Co-N_x_, pyridinic N, pyrrolic N, and graphitic N. According to previous studies, although low-stability organic nitrogen species are largely removed during pyrolysis, nitrogen in carbonaceous precursors can be transformed into more thermally stable pyridinic and graphitic nitrogen configurations at elevated temperatures, which can serve as anchoring environments for isolated metal species [39]. In addition, cobalt phthalocyanine (CoPc) used in this work provides a pre-coordinated cobalt–nitrogen molecular precursor, which is also favorable for the reconstruction of Co-N_x_ moieties during high-temperature pyrolysis. Therefore, the N 1*s* feature near 399.4 eV should be understood as spectroscopic support for the presence of Co-N_x_ species rather than standalone proof of a specific coordination structure. In this work, magnetic measurements were not performed, and therefore no magnetic-property-based claim is made.

The high-resolution Co 2*p* spectrum (Figure 2f) exhibits a characteristic Co 2*p*_3/2_/Co 2*p*_1/2_ doublet with obvious shake-up satellite peaks, while no metallic Co signal is observed. These XPS features are consistent with previous reports of Co-N_x_ single-atom sites on nitrogen-doped carbon supports, in which an N 1*s* component near 399~400 eV and Co 2*p* signals with obvious shake-up satellites are commonly assigned to cationic Co-N_x_ moieties rather than metallic Co nanoparticles [40]. Combined with the absence of crystalline Co-related reflections in XRD, these results collectively provide strong evidence that cobalt is predominantly stabilized in an atomically dispersed Co-N_x_-like coordination environment rather than as metallic cobalt nanoparticles. However, due to the limitations of the characterization results, this article believes that the atomically dispersed Co-N_x_ sites constitute the main cobalt configuration in SACo-PC-6, but the presence of trace sub-nano cobalt species cannot be completely ruled out.

**Figure 3:**
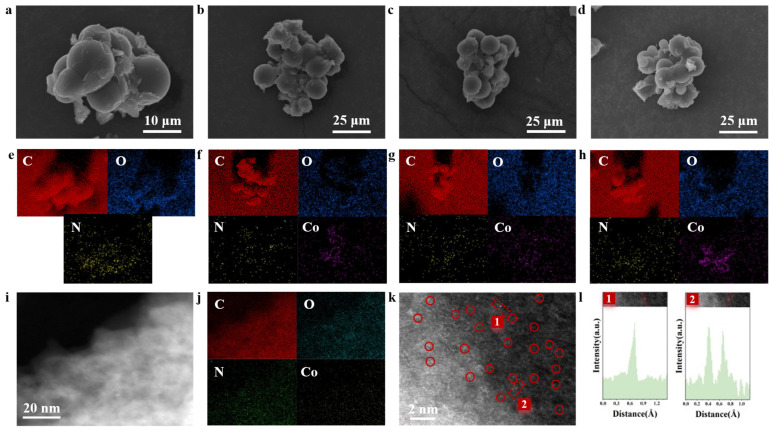
Morphological characterization of PC-Zn and SACo-PC-X: (**a**) SEM image of PC-Zn; (**b**) SEM image of SACo-PC-2; (**c**) SEM image of SACo-PC-3; (**d**) SEM image of SACo-PC-6; (**e**) EDS image of PC-Zn; (**f**) EDS image of SACo-PC-2; (**g**) EDS image of SACo-PC-3; (**h**) EDS image of SACo-PC-6; (**i**) TEM image of SACo-PC-6; (**j**) Mapping image of SACo-PC-6; (**k**) Aberration corrected HAADF-STEM image of SACo-PC-6; (**l**) Intensity profiles from the atomic sites 1 and 2. In (**k**), the red circles indicate the isolated bright spots, while the red numbered boxes 1 and 2 mark the selected regions corresponding to the intensity profiles in (**l**). The colors in the EDS elemental mappings represent the distributions of the elements indicated in each panel.

SEM images in Figure 3a–d reveal that both the PC-Zn precursor and the SACo-PC-X catalysts exhibit a uniform microspherical morphology, with diameters ranging from 2 to 5 μm. The surfaces of these microspheres are characterized by a rough texture, replete with abundant pores and cavities. This porosity is primarily attributed to the volatilization of Zn species and the release of gaseous by-products during pyrolysis. This rough surface structure greatly increases the specific surface area, providing many sites for the adsorption of AP decomposition intermediates on the catalyst surface. Furthermore, high-magnification SEM observations disclose that it was composed of many small spheres stacked together. On the surface of these spheres, we could observe a porous structure (Figure S1, Supporting Information). The enlarged SEM images provided in Figure S1 show this pore-like surface morphology more clearly, and this structural feature significantly facilitated the rapid transmission of electrons.

To further visualize the spatial distribution of the constituent elements, EDS elemental mapping was employed. As depicted in Figure 3e–h, the elements C, N, and O are homogeneously distributed throughout the entire carbonaceous framework, effectively corroborating the successful doping of nitrogen into the carbon lattice. Of significance is the Co signal (represented by pink dots), which exhibits a highly uniform dispersion across the selected region, devoid of any localized agglomeration or distinct hotspots. This observation is in excellent agreement with the XRD results, confirming the macroscopic homogeneity of Co species at the micrometer scale.

To elucidate the precise configuration of Co species at the atomic scale, the SACo-PC-6 catalyst was extensively characterized using TEM and AC-HAADF-STEM. TEM images and elemental mapping results are presented in Figure 3i,j, wherein dispersed bright signals are clearly observed on the carbon substrate. No clusters or nanoparticles were detected, suggesting that the Co species are likely dispersed at the atomic level. Theoretically, the imaging contrast in High-Angle Annular Dark-Field (HAADF) mode is governed by the atomic number (Z), being approximately proportional to Z^2^. As illustrated in Figure 3k, numerous isolated bright spots highlighted by red circles are clearly distinguished against the darker background of the carbon support. Given that the atomic number of Co (Z = 27) is substantially higher than those of C (Z = 6) and N (Z = 7), these bright spots can reasonably be assigned to atomically dispersed Co species. The absence of discernible nanoparticles or clusters larger than 1 nm within the examined field of view, together with the isolated bright spots observed by AC-HAADF-STEM, supports atomically dispersed Co species as the predominant cobalt configuration on the porous carbon matrix [41]. Furthermore, as depicted in Figure 3l, the intensity profile of Region 1 exhibits a single sharp peak, consistent with an isolated atomic center with relatively high electron density along the scan path. The profile of Region 2 displays two separated peaks with an inter-peak distance of approximately 0.27 nm, suggesting the presence of two spatially separated Co centers rather than a larger aggregated particle. Collectively, these observations support the predominance of atomically dispersed Co species, although the possible presence of trace sub-nanometric cobalt species outside the examined regions cannot be completely excluded.

### 2.2. Catalytic Effect of SACo-PC-X on the Thermal Decomposition of AP

**Figure 4:**
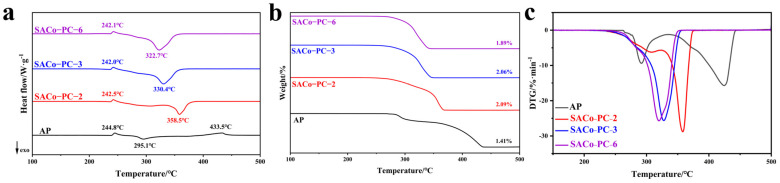
Decomposition behavior of AP and SACo-PC-X in N_2_ atmosphere: (**a**) DSC images of decomposition; (**b**) TG diagrams of decomposition; (**c**) DTG diagram of decomposition. The downward arrow in (**a**) indicates the exothermic direction.

As illustrated in Figure 4, the catalytic activity of SACo-PC-X toward the thermal decomposition of AP was systematically investigated using differential scanning calorimetry (DSC) and thermogravimetric (TG) analysis. 

The decomposition of AP is generally divided into LTD and HTD. It should be noted that proton transfer between NH_4_^+^ and ClO_4_^−^ is not itself a direct mass-loss process, but rather an initial chemical step that generates NH_3_ and HClO_4_-related species. The subsequent desorption, diffusion, and further reactions of these species are responsible for the release of gaseous products during LTD [42,43,44]. In contrast, HTD corresponds to a more extensive and rapid decomposition stage, in which NH_3_/HClO_4_-derived reactive species undergo further acid–base reactions, oxidation–reduction conversion, and transformation into nitrogen-, oxygen-, and chlorine-containing gaseous products, accompanied by concentrated heat release. Therefore, AP decomposition should be considered as a coupled multiphase process involving proton transfer, gas release, interfacial reactions, and redox conversion, rather than a mass-loss process caused solely by proton transfer. After the introduction of SACo-PC-X, the atomically dispersed Co-N_x_ sites and the porous carbon framework may jointly regulate AP decomposition. The porous structure can facilitate the diffusion and release of NH_3_/HClO_4_-derived gaseous species, while the Co-N_x_ sites may serve as interfacial catalytic centers for the adsorption and activation of reactive species, thereby promoting electron transfer and subsequent redox conversion [45,46]. This catalytic effect weakens the separation between LTD and HTD and leads to the gradual merging of the two decomposition stages into a single concentrated exothermic process, as evidenced by the DSC results.

The DSC curve of pristine AP exhibits three characteristic thermal events: an endothermic peak at 244.8 °C, corresponding to the orthorhombic-to-cubic phase transition, followed by a low-temperature decomposition (LTD) exothermic peak at 295.1 °C and a high-temperature decomposition (HTD) exothermic peak at 433.5 °C. Upon the integration of SACo-PC-X catalysts, the thermal decomposition profile of AP is dramatically altered. As the cobalt loading increases, the LTD peak gradually diminishes and merges with the HTD region, while the HTD peak temperature undergoes a pronounced downward shift. Specifically, the temperature for HTD decreases to 358.5 °C for SACo-PC-2 and 330.4 °C for SACo-PC-3. Most notably, the optimized SACo-PC-6 sample triggers a single, intense, and sharp exothermic peak at only 322.7 °C. Compared to pristine AP, SACo-PC-6 achieves a remarkable reduction in the HTD temperature by 110.8 °C. For comparison with a conventional cobalt oxide catalyst, AP mixed with 5 wt% micron-sized Co_3_O_4_ was also tested by DSC (Figure S3, Supporting Information). The HTD peak of AP shifts from 433.5 °C to 346.2 °C after the addition of micron-sized Co_3_O_4_, whereas SACo-PC-6 further decreases the peak temperature to 322.7 °C under the same catalyst loading. This comparison suggests that the atomically dispersed Co-N_x_ sites anchored on the porous carbon framework promote AP decomposition more effectively than conventional micron-sized cobalt oxide. Considering that the cobalt content in the AP/SACo-PC-6 mixture is less than 0.2%, the enhanced catalytic effect also highlights the high atomic utilization efficiency of the Co-N_x_ single-atom sites. The results from ICP analysis showed that the content of Co in the system was less than 0.2%, substantially lowering the energy barrier required for the reaction to proceed.

As evidenced by the TG and DTG profiles (Figure 4b,c), pristine AP undergoes a sluggish and prolonged mass loss process spanning a broad temperature interval. In stark contrast, the incorporation of SACo-PC-6 causes the mass loss stage to become remarkably abrupt, characterized by an exceptionally steep slope. The maximum mass loss rate—indicated by the magnitude of the DTG peak—increases substantially, while the final solid residue is reduced to a negligible level (~1.89 wt%). These observations collectively demonstrate that the SACo-PC-6 catalyst not only lowers the decomposition temperature but also significantly accelerates the reaction kinetics, effectively facilitating the near-complete decomposition of AP. To further examine whether the original SACo-PC-X structure can promote AP decomposition before obvious oxidative destruction of the catalyst framework, additional thermal-stability and isothermal TG measurements were performed. To further clarify whether SACo-PC-X can promote AP decomposition while maintaining its original catalyst framework, additional thermal-stability and isothermal TG measurements were performed. As shown in Figure S4, PC-Zn and SACo-PC-X show no obvious mass loss around 260 °C under an oxidative atmosphere, indicating that the carbon framework is largely preserved at this temperature. On this basis, isothermal TG tests of AP, PC-Zn/AP, and SACo-PC-X/AP were conducted at 260 °C (Figure S5). Pristine AP and PC-Zn/AP show only limited mass loss, whereas SACo-PC-X/AP exhibits much more pronounced decomposition. In particular, SACo-PC-6/AP shows the lowest residual mass after 50 min. These results indicate that the Co-N_x_-containing SACo-PC-X structure can already accelerate AP decomposition at 260 °C, before obvious oxidative degradation of the carbon framework occurs. Therefore, the enhanced catalytic behavior is more reasonably attributed to the original Co-N_x_-containing porous carbon structure rather than to secondary products generated from catalyst decomposition. Pristine AP and PC-Zn/AP show only limited mass loss under this condition, whereas SACo-PC-X/AP exhibits much more pronounced decomposition. In particular, SACo-PC-6/AP shows the lowest residual mass, suggesting that the Co-N_x_-containing porous carbon structure can already accelerate AP decomposition at a relatively low temperature.

### 2.3. Gas-Product and Kinetic Analysis of AP Mixed with 5 wt% SACo-PC-X 

To elucidate the underlying catalytic mechanism, a comprehensive investigation was conducted, encompassing non-isothermal kinetic analysis and evolved gas analysis. 

**Figure 5:**
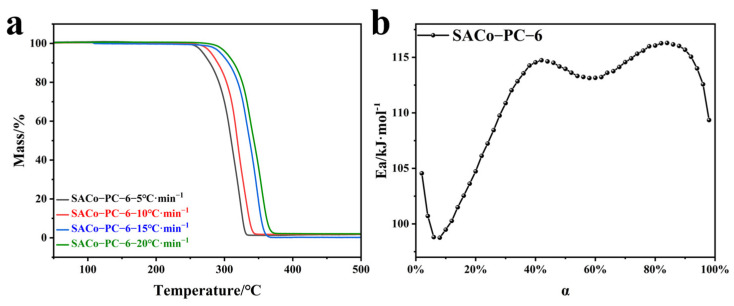
Dynamics analysis of SACo-PC-6 catalyzed AP: (**a**) TG of SACo-PC-6 catalyzed AP at different heating rates; (**b**) Variation of activation energy of SACo-PC-6 catalyzed AP with reaction degree.

As illustrated in Figure 5, the apparent activation energy (Ea) was determined by isoconversional analysis using DSC data collected at different heating rates (see Section 2 of the Supporting Information for the detailed calculation method [47,48]). According to the ICTAC recommendations, a clear dependence of Ea on conversion generally suggests that the decomposition does not follow a single-step mechanism, but rather involves multiple overlapping steps or changes in the rate-controlling contribution during the reaction progress [49,50]. Such behavior has also been reported for AP itself. For example, Vyazovkin and Wight showed that the effective activation energy of cubic AP varies with conversion and increases again at high conversion, which was interpreted in connection with the complex interplay of decomposition and sublimation processes [51]. Therefore, the conversion-dependent Ea values observed for the SACo-PC-6/AP system in this work support the view that the catalytic decomposition remains a multi-step process rather than a single elementary reaction. The corresponding kinetic results for pristine AP and the SACo-PC-2/AP and SACo-PC-3/AP systems are provided in Figure S6 [52] and Figure S7 [53,54], respectively, further confirming the conversion-dependent feature of AP decomposition.

The calculation results of the activation energy of AP indicate that the activation energy shows a trend of first decreasing and then increasing throughout the entire process (the early data points may deviate far due to errors), and the reaction degree α position of the extreme point corresponds exactly to the stagnation stage of AP’s own decomposition. When considered in conjunction with earlier research findings, it can be concluded that this signifies the transition of AP’s decomposition rate step from self-decomposition to NH_3_ and HClO_4_ to their desorption on the AP surface, entering LTD, leading to a subsequent alteration in the rate step and an overall increase in activation energy. The addition of SACo-PC-6 results in a diminution of the catalytic effect of the initial catalyst, thereby maintaining the trend of activation energy change. Subsequently, the adsorption and catalytic conversion of NH_3_ and HClO_4_ by the metal active sites on the catalyst surface renders these gases no longer the determining steps, thus causing the activation energy to begin to rise. The overall trend of activation energy change is consistent, but the appearance of the extremum point is earlier.

**Figure 6:**
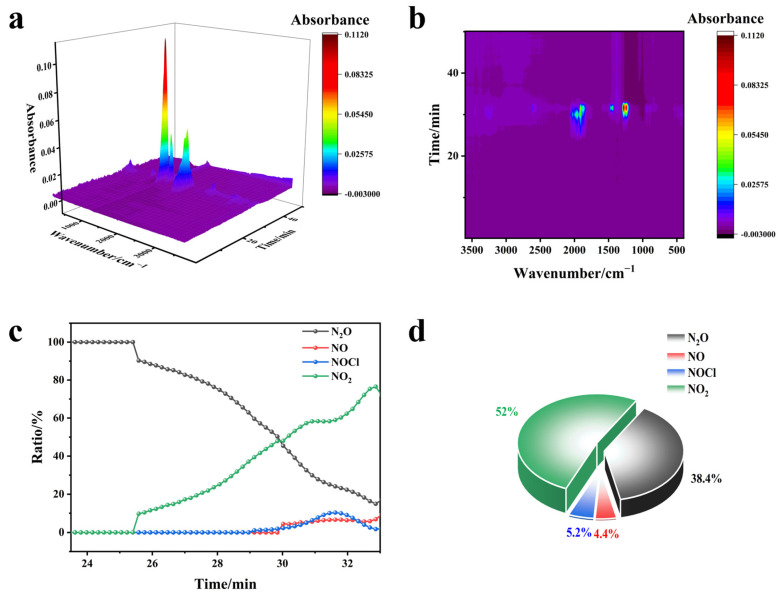
Gas composition analysis of SACo-PC-6-catalyzed AP decomposition: (**a**) 3D TG-IR diagram; (**b**) 2D TG-IR mapping diagram; (**c**) time-dependent relative gas composition; (**d**) overall gas composition diagram.

The evolution of gaseous products during AP decomposition catalyzed by SACo-PC-6 was systematically investigated via TG-IR analysis (Figure 6). The evolution patterns of major gaseous products, including N_2_O, NO, NO_2_ and NOCl, during the decomposition of pristine AP (Figure S9, Supporting Information). The 3D and mapping TG-IR spectra (Figure 6b) reveal that the gaseous species are primarily released between 25 and 35 minutes, with characteristic signals corresponding to N_2_O, NO, NO_2_ and NOCl. 

Quantitative results show that NO_2_ (52.0%) and N_2_O (38.4%) are the predominant nitrogen-containing products in terms of overall relative composition, with the relative proportion of NO_2_ reaching 50.6% during the most intense decomposition stage (Figure 6c,d). Additionally, the gaseous product evolution and quantitative analysis for the SACo-PC-2 and SACo-PC-3 systems further confirm the regulated electron transfer and accelerated oxidation pathways facilitated by Co-N_x_ sites (Figures S9 and S10, Supporting Information).

**Figure 7:**
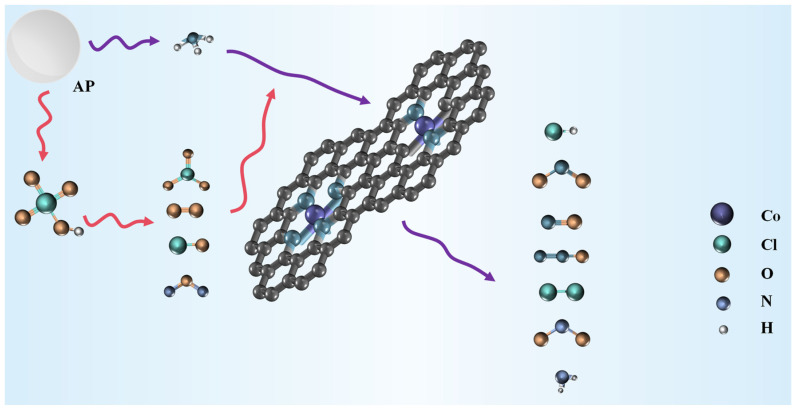
Catalytic mechanism of SACo-PC-6. The arrows indicate the proposed sequence of AP decomposition, migration and activation of AP-derived intermediates over SACo-PC-6, and the subsequent formation of gaseous products.

The variation in gaseous product distribution is important because it reflects changes in the decomposition pathway of AP rather than a simple shift in decomposition temperature [55,56]. For AP-based propellants, gaseous products are directly related to gas generation, heat release, flame chemistry, and the subsequent combustion process [57]. Therefore, the altered NO_2_/N_2_O/NO/NOCl distribution observed after introducing SACo-PC-X further suggests that Co-N_x_ sites regulate the oxidation pathway of nitrogen-containing intermediates and the conversion of chlorine-containing species. It should also be noted that thrust and specific impulse are determined by the complete propellant formulation and motor operating conditions. Therefore, the present TG-IR results do not directly prove an improvement in thrust, but they provide mechanistic evidence that SACo-PC-X changes the gas-evolution pathway of AP decomposition, which is relevant to propellant combustion behavior. In addition to the programmed-heating TG-IR measurements, isothermal TG-IR analysis was further carried out at 260 °C to monitor the gas-evolution behavior of SACo-PC-X/AP during low-temperature decomposition (Figures S11–S13). Characteristic gaseous signals are detected for SACo-PC-2/AP, SACo-PC-3/AP, and SACo-PC-6/AP under the isothermal condition, demonstrating that SACo-PC-X can promote the release and conversion of AP decomposition intermediates at 260 °C. Notably, SACo-PC-6/AP exhibits the strongest infrared signals among the tested samples, which is consistent with its lowest residual mass in the isothermal TG test. These results further confirm that SACo-PC-6 has the strongest low-temperature catalytic activity within the SACo-PC-X series. Based on the above DSC, kinetic, and TG-IR results, a possible catalytic decomposition mechanism of AP over SACo-PC-6 is proposed and schematically illustrated in Figure 7.

Based on the above DSC, kinetic, and TG-IR results, a possible catalytic decomposition mechanism of AP over SACo-PC-6 is proposed and schematically illustrated in Figure 7. In this mechanism, the hierarchical porous carbon framework facilitates the diffusion and release of NH_3_/HClO_4_-derived gaseous species and improves the accessibility of catalytic sites. Meanwhile, the atomically dispersed Co-N_x_ moieties are proposed to act as interfacial active centers that promote the adsorption, activation, and redox conversion of reactive decomposition species. As a result, the separation between LTD and HTD is weakened, the decomposition process becomes more concentrated, and the gaseous product distribution is altered. This synergistic effect between the porous carbon framework and Co-N_x_ sites accounts for the enhanced catalytic activity of SACo-PC-6 toward AP thermal decomposition.

## 3. Materials and Methods

### 3.1. Materials

All reagents were purchased from commercial sources and used directly without further purification. The rice used in this study was leftover cooked rice collected from a school cafeteria, and zinc chloride (ZnCl_2_, AR), hydrochloric acid (HCl) 36.0–38.0%, and ethanol (C_2_H_5_OH, AR) used in this study were all purchased from Sinopharm Chemical Reagent Co., Ltd. (SCRC, Shanghai, China). Cobalt phthalocyanine (CoPc, C_32_H_16_N_8_Co, AR) was purchased from Shanghai Aladdin Biochemical Technology Co., Ltd. (Shanghai, China). Distilled water is self-made in the laboratory.

### 3.2. Synthesis

Preparation of Fermented Rice: First, leftover cooked rice was collected from the canteen. Then, 500 mL of rice was placed in a bowl, mixed with some yeast powder and an appropriate amount of water, and stirred by hand to mix uniformly. Subsequently, the mixed rice was sealed and fermented at 30 °C for 3 days to obtain the product.

Preparation of N-doped Porous Carbon (PC-Zn): 6 g of fermented rice was mixed with 60 mL of deionized water and placed in a 100 mL Teflon-lined autoclave, then kept at 180 °C for 24 h. This hydrothermal step was adapted from literature-reported hydrothermal carbonization procedures for biomass-to-hydrochar conversion [58]. This yielded the N-doped porous carbon precursor. Subsequently, the precursor was mixed with ZnCl_2_ at a mass ratio of 1:2 and placed in a tube furnace. Under a N_2_ atmosphere, the temperature was raised to 700 °C at a heating rate of 5 °C·min^−1^ and maintained for 2 h. The ZnCl_2_-activated N-doped porous carbon was denoted as PC-Zn.

Preparation of SACo-PC-X: The prepared PC-Zn was mixed with CoPc at different mass ratios. The uniform powder was then transferred into a porcelain boat and placed in a tube furnace. Under a N_2_ atmosphere, it was heated to 800 °C at a rate of 5 °C·min^−1^ and held for 2 h. The resulting single-atom cobalt-loaded carbon-nitrogen catalyst powder was denoted as SACo-PC-X.

Preparation of AP/catalyst mixture: Weigh an appropriate amount of catalyst and mix it with AP. Add an appropriate amount of ethanol, perform ultrasonic dispersion for 10 min, and then dry at 40 °C for 5 h. Subsequently, thoroughly mix the dried powder until there is no obvious color difference in each region. Unless otherwise specified in this text, the mass fraction of the catalyst is 5%.

The characterizations are shown in Supporting Information.

## 4. Conclusions

In this work, an in situ pyrolysis strategy integrating “Zn-volatilization-induced pore formation” and “nitrogen-anchoring” was developed to synthesize biomass-derived nitrogen-doped porous carbon-supported single-atom cobalt catalysts Co-SACs. Comprehensive structural characterization suggests that atomically dispersed Co-N_x_-like moieties constitute the predominant cobalt configuration within the carbon framework, although the possible presence of trace sub-nanometric cobalt species cannot be completely excluded. Notwithstanding this limitation in the structural assignment, the resulting catalysts demonstrate exceptional catalytic activity toward the thermal decomposition of AP. Specifically, the optimized catalyst achieves a dramatic reduction in the HTD peak temperature by 110.8 °C and significantly augments the apparent exothermic heat release. More importantly, the cobalt metal content in the system is only less than 0.2%, which fully demonstrates the significant improvement in the atomic utilization efficiency of the catalyst. Mechanistic investigations suggest that atomically dispersed Co-N_x_ sites may facilitate electron-transfer processes and participate in regulating AP decomposition pathways. This synergy accelerates the oxidation of NH_3_ and the deep conversion of NO_x_ intermediates, ultimately optimizing the reaction pathways and product distribution. This work not only offers a cost-effective and environmentally benign synthesis paradigm for the development of high-performance burning rate catalysts for AP-based propellants but, more significantly, provides atomic-scale insights into the unique catalytic mechanisms of single-atom active sites during the thermal decomposition of energetic materials. These findings establish a robust theoretical and experimental framework for the future rational design of highly efficient and stable single-atom catalysts (SACs). The development of this catalyst offers novel insights for the design of AP catalysts.

## Data Availability

Data will be made available on request.

## Supplementary Materials

The following supporting information can be downloaded at: https://www.mdpi.com/article/10.3390/ijms27135964/s1.
