# Supplementary material for: Biochar-Based Single-Atom Cobalt Catalyst for Efficient Thermal Decomposition of Ammonium Perchlorate: Preparation, Performance and Mechanism
Source: Int J Mol Sci. 2026 Jul 2;27(13):5964. doi: 10.3390/ijms27135964 (PMC13362475; doi:10.3390/ijms27135964)
Supplement: Supplementary file 1 [file ijms-27-05964-s001.zip › ijms-4363131-supplementary.pdf]

Supporting Information

Biochar-Based Single-Atom Cobalt Catalyst for Efficient Thermal Decomposition of Ammonium Perchlorate: Preparation, Performance and Mechanism

Yixin Liu [1,†], Xiaolin Tang [1,†], Bin Zhang [2], Yuming Zhou [1], Junyu Li [1], Zeyu Zheng [1], Yifu Zhang [1,3,*], Yanfen Huang [4] and Chi Huang [1,*]

[1] College of Chemistry and Molecular Sciences, Wuhan University, Wuhan 430072, China; 2024282030194@whu.edu.cn (Y.L.); winters@whu.edu.cn (X.T.); yumingzhou@whu.edu.cn (Y.Z.); junyuli@whu.edu.cn (J.L.); zhengzeyu@whu.edu.cn (Z.Z.)

[2] System Design Institute of Hubei Aerospace Technology Academy, Wuhan 430040, China; zhangbin8110@163.com

[3] Hubei Key Laboratory of Radiation Chemistry and Functional Materials, School of Nuclear Technology and Chemistry & Biology, Hubei University of Science and Technology, Xianning 437100, China

[4] School of Chemistry and Chemical Engineering, Wuhan University of Science and Technology,
Wuhan 430081, China; huangyanfen@wust.edu.cn

* Correspondence: yfzhang2023@whu.edu.cn (Y.Z.); chihuang@whu.edu.cn (C.H.)
† These authors contributed equally to this work.

Section 1. Instruments

Raman reflectance spectroscopy (Raman)

The Raman test was conducted using the RENISHAW inVia™ InSpect confocal Raman microscope.

Powder X-ray diffraction (PXRD)

PXRD measurement was recorded on a Shimadzu XRD-6100 diffractometer from Japan. The diffractometer was tested under ambient temperature and pressure. The specific experimental conditions are as follows: the target material is a copper target, the wavelength of X-rays is 0.1542 nm, the scanning rate is $10^{o}\cdot min^{-1}$, and the scanning angle range is 10~80º.

X-ray photoelectron spectroscopy (XPS)

XPS was recorded on a Thermo Fisher K-Alpha X-ray photoelectron spectrometer. The light source used is an aluminum target with HV=1486.6 eV, and the analysis area is 400 μm. Before peak splitting, pollution carbon correction BE=284.8 eV is performed on the test results.

Gas adsorption-desorption test (BET)

BET testing was measured on a Physichem Instruments ipore 400 equipped with a liquid nitrogen bath (77 K). Ultra-high purity grade N_2 was used for the adsorption experiments. N_2 adsorption–desorption isotherms were measured at 77 K. The specific surface area was calculated by the BET method, and the pore-size distribution was derived from the N_2 sorption data using the NLDFT equilibrium model. The isotherm and hysteresis-loop types were assigned according to the IUPAC technical report.

Inductively Coupled Plasma Optical Emission Spectrometer (ICP-OES)

The elemental analysis was recorded on an Agilent 5800 ICP-OES spectrometer

from USA. The specific experimental conditions are as follows: the RF power is 1.20 kW, the plasma gas flow is 12.00 $L \cdot min^{-1}$, the auxiliary gas flow is 1.00 $L \cdot min^{-1}$, the nebulizer gas flow is 0.70 $L \cdot min^{-1}$, and the pump speed is 12 rpm.

Scanning electron microscopy (SEM)

SEM images were taken using Zeiss Merlin Compact, and before shooting, the sample was treated with platinum plating to enhance its conductivity, with an acceleration voltage of 5 kV.

Energy Dispersive Spectroscopy (EDS)

EDS analysis and elemental mapping were performed using a Zeiss GeminiSEM 500 field emission scanning electron microscope. The measurements were carried out with an acceleration voltage of 5 kV (or your specific test voltage) to ensure optimal signal detection while maintaining surface sensitivity. The system's high resolution, capable of reaching 0.5 nm at 15 kV and 0.9 nm at 1 kV, provided precise localized elemental distribution data for the samples.

Transmission electron microscopy (TEM)

TEM images were taken using a JEM-2100 transmission electron microscope, with an acceleration voltage of 200 kV. TEM elemental analysis mapping image captured on Talos F200X G2 transmission electron microscope.

Ball aberration correction transmission electron microscope (AC-TEM)

The atomic resolution high-angle annular dark-field scanning transmission electron microscopy (HAADF-STEM) images were acquired on a JEOL JEM-ARM300F2 (GRAND ARM2) spherical aberration-corrected transmission electron microscope. The instrument was operated at an accelerating voltage of 300 kV. The sample was dispersed on a microgrid for observation.

Thermogravimetric analysis

TG-DSC is used to analyze the quality changes and heat absorption and release of samples under a programmed heating system. In this article， TG-DSC analysis from 30-500 ℃ were tested on a German NETZSCH STA-2500 synchronous thermal analyzer using a 10 ℃·min^{-1} heating rate. For AP or AP/catalyst mixed samples, the testing atmosphere is nitrogen (Except for special markings).

TG-IR analysis

TG-IR testing is used to analyze the decomposition products of AP or AP/catalyst samples and for relative quantitative comparison. The carrier gas flow rate decreased to 20 mL min^{-1}. The IR instruments are Thermo Fisher and IS-10 Fourier transform infrared spectrometers.

Section 2. Activation Energy Calculation

This paper calculates the kinetics based on TG data, assuming that the total mass lost by the sample during the TG process is α=1, and then calculates α based on the mass loss at different temperatures to obtain the α - T curve. In order to further obtain the T value under a specific α, quadratic interpolation is used to process α-T.

$$f(x_0) = af_{l-1} + bf_l + cf_{l+1}$$

$$a = \frac{(x_0 - x_l) \times (x_0 - x_{l+1})}{(x_{l-1} - x_l) \times (x_{l-1} - x_{l+1})}$$

$$b = \frac{(x_0 - x_{l-1}) \times (x_0 - x_{l+1})}{(x_l - x_{l-1}) \times (x_l - x_{l+1})}$$

$$c = \frac{(x_0 - x_{l-1}) \times (x_0 - x_l)}{(x_{l+1} - x_{l-1}) \times (x_{l+1} - x_l)}$$

After obtaining the α-T curve, the E_a values of the reaction at different α values were obtained using the OZAWA method.

$$\log\beta = -\frac{0.4567\Delta E}{RT_m} - 2.315 + \log\frac{A\Delta E}{R} - \log G(x_m)$$

Section 3. Supplementary Figures

SEM images reveal that both PC-Zn and SACo-PC-X exhibit uniform microspherical morphology with rough surfaces and abundant pores, facilitating intimate contact with AP particles. This spherical architecture features a rough surface with abundant pores, providing sufficient contact area for catalytic reactions and accelerating electron transport.

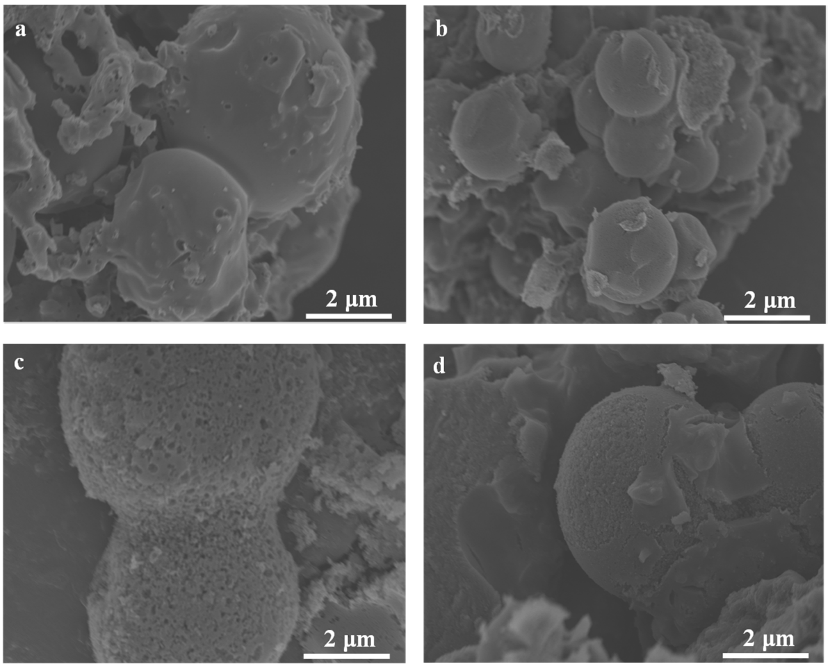

Figure S1. Morphological characterization of PC-Zn and SACo-PC-X; (a) SEM image of PC-Zn; (b) SEM image of SACo-PC-2; (c) SEM image of SACo-PC-3; (d) SEM image of SACo-PC-6.

N_2 adsorption-desorption isotherms and pore size distribution confirm the well-developed mesoporous structure of PC-Zn (pore size ~3–5 nm), effectively promoting the diffusion of reactants and intermediates within the pores. This hierarchical porous architecture effectively promotes the diffusion of reactants and intermediates during AP decomposition.

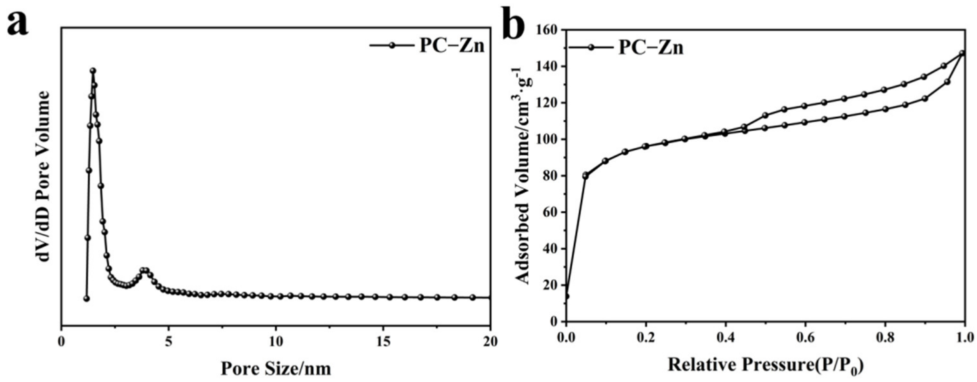

Figure S2. Structural characterization of PC-Zn: (a) N_2 sorption isotherms of PC-Zn at 77 K; (b) Apparent pore-size distribution of PC-Zn derived from N_2 sorption data

A comparative DSC test was performed to evaluate the catalytic effect of SACo-PC-6 relative to a conventional micron-sized cobalt oxide catalyst. As shown in Figure S3, pristine AP exhibits a phase-transition endothermic peak at 245.9°C, a low-temperature decomposition peak at 297.1°C, and a high-temperature decomposition peak at 433.5°C. After the addition of 5 wt% micron-sized Co_3O_4, the main decomposition peak shifts to 346.2°C, indicating that micron-sized cobalt oxide can promote AP decomposition. In comparison, SACo-PC-6 further decreases the main decomposition peak to 322.7°C, demonstrating its stronger catalytic effect toward AP thermal decomposition.

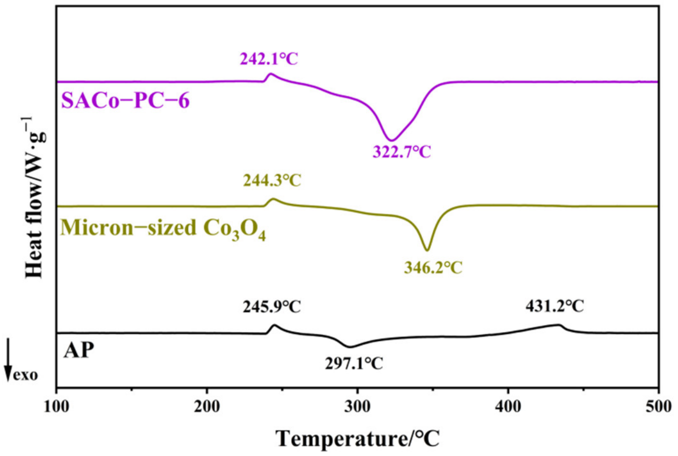

Figure S3. DSC curves of pristine AP, AP mixed with 5 wt% micron-sized Co_3O_4, and AP mixed with 5 wt% SACo-PC-6.

The thermal stability of PC-Zn and SACo-PC-X was evaluated under an oxidative atmosphere to clarify whether the catalyst framework remains stable at the temperature used for the subsequent isothermal test. As shown in Figure S4, PC-Zn and SACo-PC-X show no obvious mass loss around 260°C, indicating that the catalyst structure is largely preserved at this temperature. The main mass loss occurs at higher temperatures and can be mainly attributed to the oxidative decomposition of the biomass-derived carbon framework.

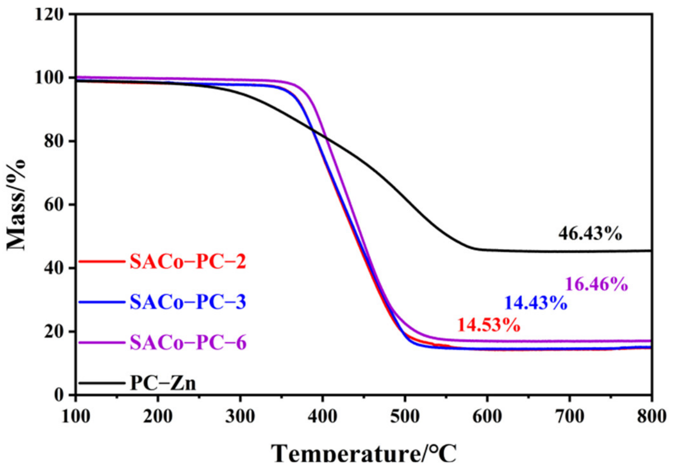

Figure S4. TG curves of PC-Zn and SACo-PC-X under an oxidative atmosphere.

Isothermal TG analysis was performed to further evaluate the low-temperature catalytic effect of SACo-PC-X on AP decomposition. As shown in Figure S5, pristine AP and PC-Zn/AP show only slight mass loss at 260°C, with residual masses of 88.75% and 90.26%, respectively. In contrast, the residual masses of SACo-PC-2/AP, SACo-PC-3/AP, and SACo-PC-6/AP decrease to 58.36%, 57.71%, and 34.54%, respectively. This result indicates that SACo-PC-X can promote AP decomposition at relatively low temperature, with SACo-PC-6 showing the strongest catalytic effect among the tested samples.

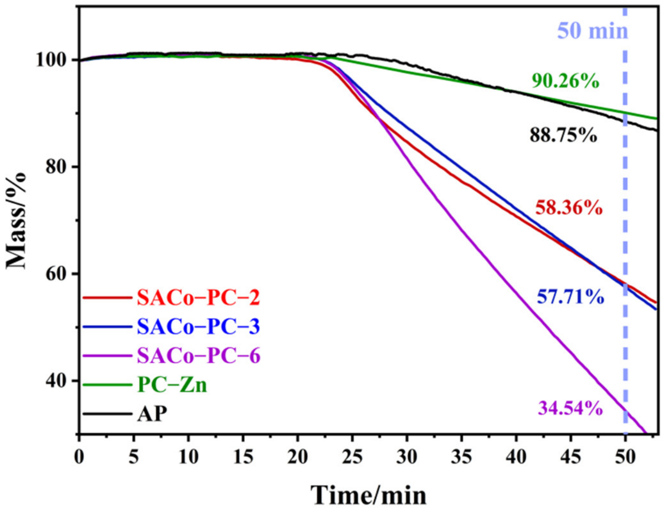

Figure S5. Isothermal TG curves of AP, PC-Zn/AP, and SACo-PC-X/AP at 260 °C.

TG and kinetic analysis show the decomposition behavior of pristine AP at different heating rate. The results indicate that the high-temperature decomposition stage of pure AP possesses a high apparent activation energy barrier, typically exceeding 160 kJ/mol.

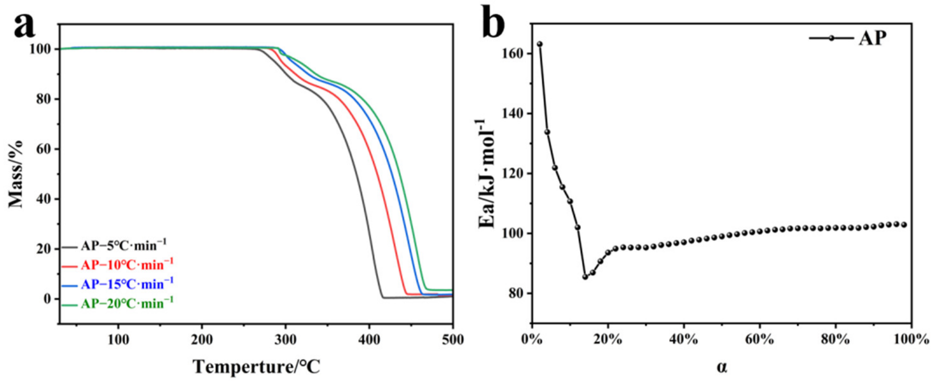

Figure S6. Dynamics analysis of AP: (a) TG of AP at different heating rates; (b) Variation of activation energy of AP with reaction degree

Kinetic analysis indicates that both SACo-PC-2 and SACo-PC-3 significantly lower the HTD temperature of AP to 358.5 °C and 330.4 °C, respectively. Comparison reveals that catalytic activity is markedly enhanced with increasing cobalt loading, evidenced by a further reduction in HTD temperature. Furthermore, the variation of activation energy with conversion reflects the complex multi-step nature of the catalytic process.

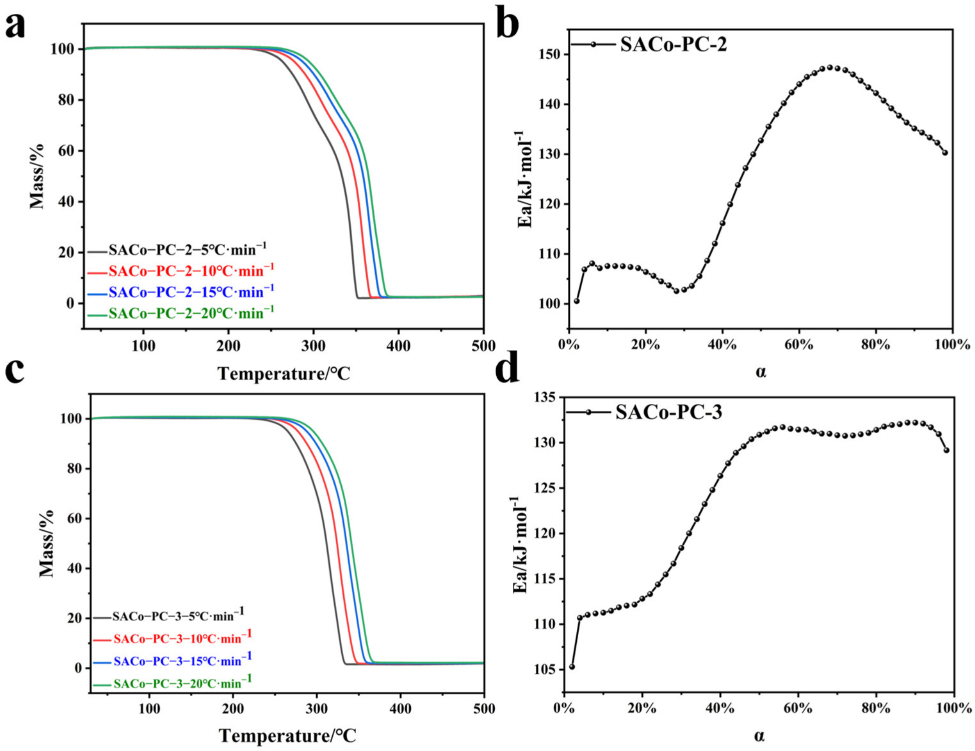

Figure S7. Dynamics analysis of SACo-PC-2 catalyzed AP and SACo-PC-3 catalyzed AP: (a) TG of SACo-PC-2 catalyzed AP at different heating rates; (b) Variation of activation energy of SACo-PC-2 catalyzed AP with reaction degree; (c) TG of SACo-PC-2 catalyzed AP at different heating rates; (d) Variation of activation energy of SACo-PC-2 catalyzed AP with reaction degree

TG-IR analysis reveals the evolution patterns of major gaseous products, including N_2O, NO, NO_2, and NOCl, during the decomposition of pristine AP.

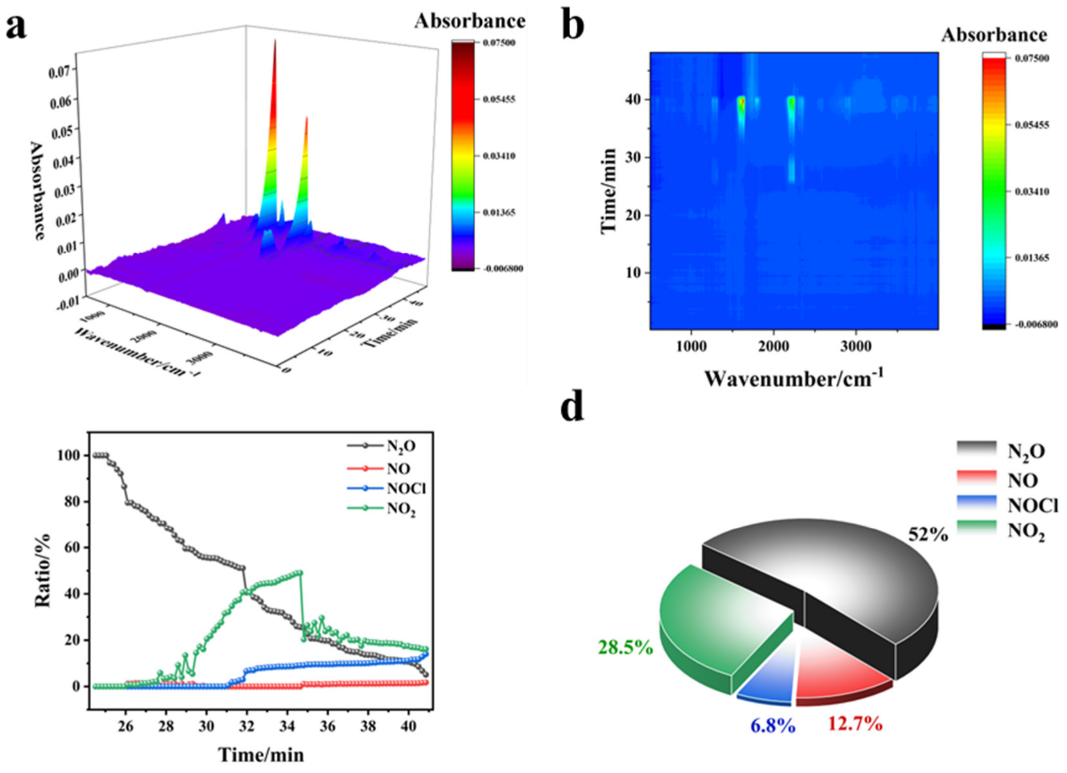

Figure S8. Gas composition analysis of AP decomposition: (a) 3D TG-IR diagram; (b) 2D TG-IR mapping diagram; (c) time-dependent relative gas composition; (d) overall gas composition diagram

Monitoring of gaseous products shows that the SACo-PC-2 catalyst alters product distribution by regulating electron transfer pathways.

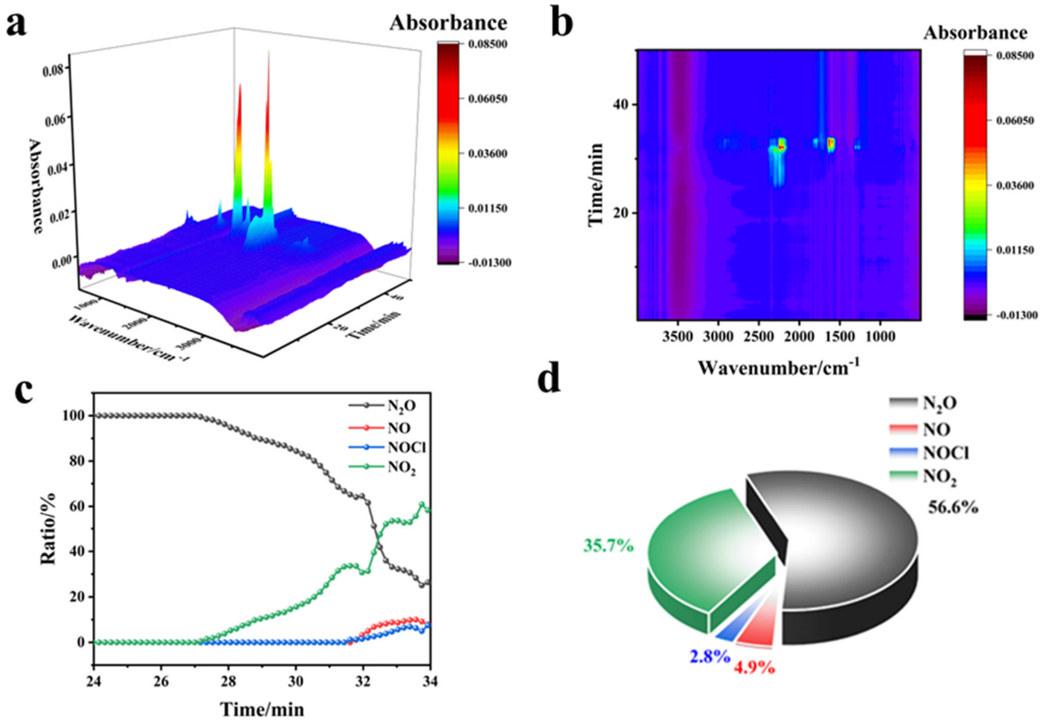

Figure S9. Gas composition analysis of SACo-PC-2 catalyzed AP decomposition: (a) 3D TG-IR diagram; (b)2D TG-IR mapping diagram; (c) time-dependent relative gas composition; (d) overall gas composition diagram

Quantitative gaseous analysis indicates that Co-Nx active sites in SACo-PC-3 accelerate the oxidation process, significantly increasing the production proportion of the exothermic product N_2O.

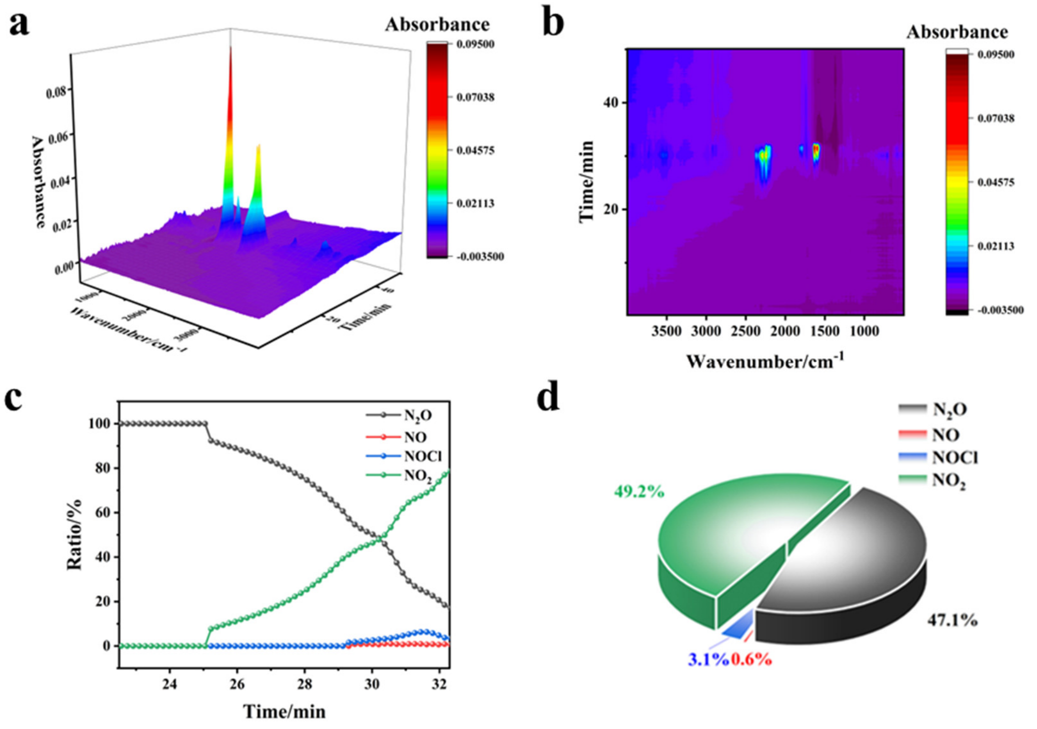

Figure S10. Gas composition analysis of SACo-PC-3 catalyzed AP decomposition: (a) 3D TG-IR diagram; (b)2D TG-IR mapping diagram; (c) time-dependent relative gas composition; (d) overall gas composition diagram

The gas-evolution behavior of SACo-PC-2/AP during low-temperature decomposition was further monitored by isothermal TG-IR analysis. As shown in Figure S11, characteristic infrared signals of gaseous decomposition products are observed, indicating that SACo-PC-2 can promote the release and conversion of AP decomposition intermediates under the isothermal condition.

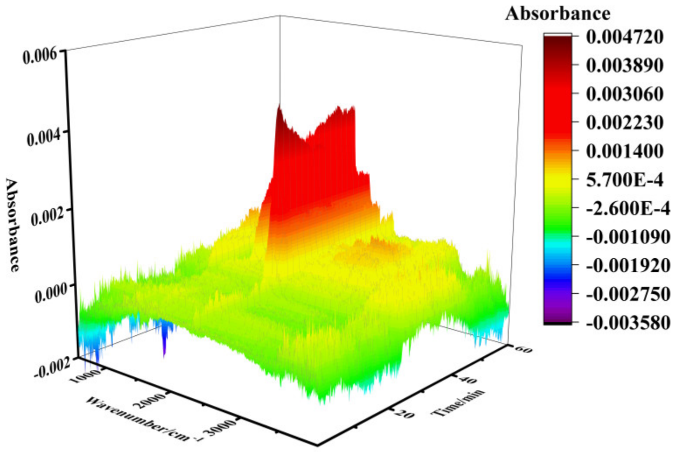

Figure S11. Isothermal 3D TG-IR analysis of SACo-PC-2/AP at 260 °C

For SACo-PC-3/AP, the isothermal TG-IR results show similar gas-evolution behavior to that of SACo-PC-2/AP. As shown in Figure S12, gaseous decomposition products are also detected under the isothermal condition, suggesting that SACo-PC-3 can promote AP low-temperature decomposition and the formation of gaseous intermediates.

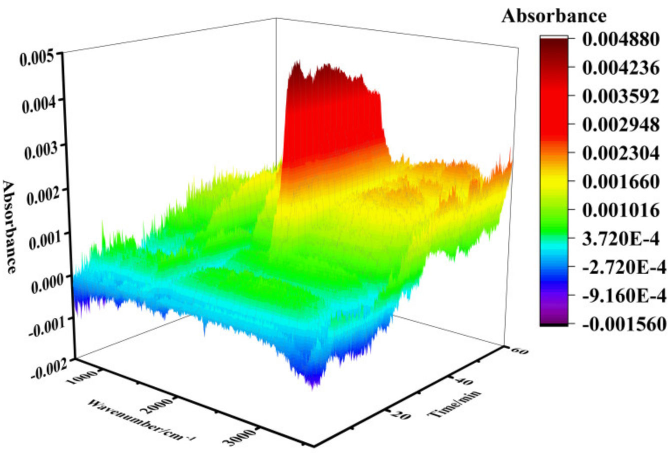

Figure S12. Isothermal 3D TG-IR analysis of SACo-PC-3/AP at 260 °C

The isothermal TG-IR spectra of SACo-PC-6/AP show stronger infrared absorption signals than those of SACo-PC-2/AP and SACo-PC-3/AP. As shown in Figure S13, this more pronounced gas release indicates that SACo-PC-6 more effectively promotes the generation and conversion of AP decomposition intermediates at 260°C. This result is consistent with the isothermal TG analysis and further supports the stronger low-temperature catalytic activity of SACo-PC-6.

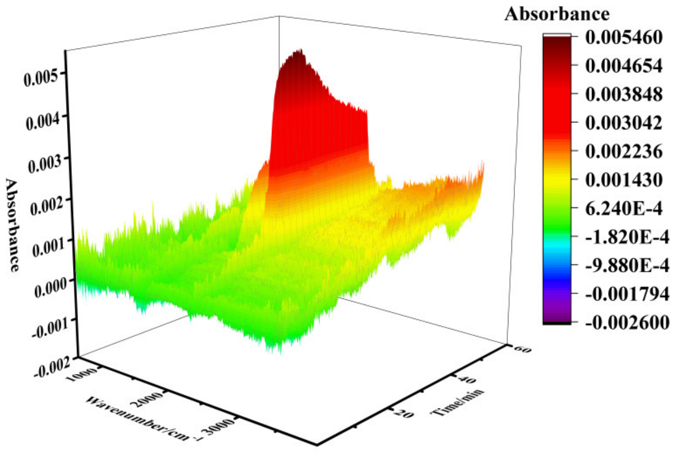

Figure S13. Isothermal 3D TG-IR analysis of SACo-PC-6/AP at 260 °C
